# Genomic Alteration Burden in Advanced Prostate Cancer and Therapeutic Implications

**DOI:** 10.3389/fonc.2019.01287

**Published:** 2019-11-22

**Authors:** Matthew J. Ryan, Rohit Bose

**Affiliations:** ^1^Medical Scientist Training Program, University of California, San Francisco, San Francisco, CA, United States; ^2^Biomedical Sciences Graduate Program, University of California, San Francisco, San Francisco, CA, United States; ^3^Department of Anatomy, University of California, San Francisco, San Francisco, CA, United States; ^4^Departments of Medicine and Urology, University of California, San Francisco, San Francisco, CA, United States; ^5^UCSF Helen Diller Family Comprehensive Cancer Center, San Francisco, CA, United States; ^6^UCSF Benioff Initiative for Prostate Cancer Research, San Francisco, CA, United States

**Keywords:** prostate cancer, tumor mutation burden, copy number alteration, structural variants, aneuploidy, mismatch repair deficiency, immune checkpoint inhibitor

## Abstract

The increasing number of patients with sequenced prostate cancer genomes enables us to study not only individual oncogenic mutations, but also capture the global burden of genomic alterations. Here we review the extent of tumor genome mutations and chromosomal structural variants in various clinical states of prostate cancer, and the related prognostic information. Next, we discuss the underlying mutational processes that give rise to these various alterations, and their relationship to the various molecular subtypes of prostate cancer. Finally, we examine the relationships between the tumor mutation burden of castration-resistant prostate cancer, DNA repair defects, and response to immune checkpoint inhibitor therapy.

## Introduction

Prostate cancer is the second-most common cancer in men worldwide (1), and advanced forms of the disease cause debilitating bone pain, pathologic fractures, and severe anemia. The era of profiling patients' tumors using next generation sequencing (NGS) has yielded both scientific and clinical advances including: the comprehensive detection of *BRCA* mutations for PARP inhibitor therapy; the identification of poorer prognosis *RB1* mutations; and the full extent of *AR* genomic alterations associated with androgen receptor signaling inhibitor (ARSI) resistance (2–6). Equally important, NGS profiling has expanded our insight beyond a handful of known loci to capture our first snapshots of the prostate tumor genome in its entirety. This raises the question: does the global burden of mutations and chromosomal structural variants reveal information beyond individual driver mutation analysis?

Here we examine the genomic alteration burden in various states of prostate cancer, a disease with a heterogenous clinical course. Next, we delve into the various mutational processes underlying those alterations and highlight associations with molecular subtypes. Finally, we evaluate how a tumor's mutation burden may help predict response to certain therapies. There are several caveats: factors beyond the tumor genome, such as the transcriptome, epigenome, and the microenvironment are undoubtedly relevant, but beyond the scope of this mini review. Secondly, the analyzed cohorts are predominantly comprised of patients of European ancestry. Finally, this review of global genomic alterations is simply designed to augment, not supersede, the relevance of individual mutations and traditional clinical parameters.

## Burden of Genomic Alterations in Different Clinical States

Tumor mutation burden (TMB) (7) is measured differently among various prostate cancer cohorts. Sometimes, it is reported as the load of non-synonymous mutations (NS) with a minimum allele frequency of 0.5–10%. Other times, it is reported as the load of any single nucleotide variants (SNVs). Some studies additionally report the rate of indels (8, 9). The TMB of unselected and usually treatment-naïve locoregional prostate adenocarcinoma cohorts typically falls between 0.94 and 1.74 NS per megabase (Mb) (Table 1). Average TMB appears to correlate with the patient's age at diagnosis (~0.5 NS/Mb for those diagnosed in their 40s vs. ~0.9 NS/Mb in their 60s) (12). Primary tumor grade is a major clinical feature and described by the Gleason score (currently being updated to the Grade Group system) (33). The SNV burden has been reported as 1.5 × higher in intermediate pattern Gleason 7 tumors vs. well-differentiated pattern Gleason 6 tumors (*p* = 1.05 × 10^−3^) (16), consistent with other reports (12). Interestingly, a small cohort of South African patients of African ancestry with high-risk locoregional disease were found to have a roughly 4-fold increase of TMB (3.0–4.7 SNVs plus indels/Mb) (Table 1) compared with a control cohort of European ancestry (23). On the other hand, a study of African-American men with primary prostate cancer had a rate of 0.83 SNVs/Mb, in line with cohorts of predominantly European-Americans (17).

**Table 1:** Tumor mutation burden (TMB) in locoregional, metastatic castration-sensitive (mCSPC), and metastatic castration-resistant (mCRPC) prostate cancer samples.

**Clinical state**	**TMB (mutations per Mb or sample)**	**Method of sequencing**	**Algorithm for somatic mutation calling**	**Cohort (number of samples)^a^**	**References**
Locoregional	0.94 NS/Mb^b^	WES	MuTect (10)	TCGA, *Cell* 2015 (*n* = 333)	(11)
	1.36 NS/Mb^c^	WES	MuTect (10)	MSKCC/DFCI, *Nature Genetics* 2018 (*n* = 1013)	(12)
	1.74 NS/Mb^c^	Gene panel (MSK-IMPACT)^d^	MuTect (10)	MSKCC, *JCO Precis Oncol* 2017 (*n* = 504)	(13)
	33 NS/sample^c^^,^ ^e^	WGS	MuTect (10)	Broad/Cornell, *Cell* 2013 (*n* = 57)	(14)
	0.53 SNVs/Mb^b^	WGS	SomaticSniper (15)	CPC-GENE, *Nature* 2017 (*n* = 477)	(16)
	0.83 SNVs/Mb^b^	WES	MuTect (10)	Cornell/Karmanos, *Cancer Discov* 2017 (*n* = 102)	(17)
	0.93 SNVs/Mb^c^	WES	Used own method	MCTP, *Nature* 2012 (*n* = 61)	(18)
	0.93 SNVs/Mb^b^	WES	VarScan (19)	PROGENY Study, *Ann Oncol* 2017 (*n* = 49)	(20)
	1.4 SNVs/Mb^b^	WES	MuTect (10)	Broad/Cornell, *Nat Genet* 2012 (*n* = 112)	(21)
	3.0–4.7 SNVs plus indels/Mb	WGS	MuTect, Strelka, VarScan (10, 19, 22)	SAPCS, *Cancer Res* 2018 (*n* = 15)	(23)
mCSPC	2.08 NS/Mb^c^	Gene panel (MSK-IMPACT)^e^	MuTect (10)	MSKCC, *JCO Precis Oncol* 2017 (*n* = 504)	(13)
mCRPC	4.02 NS/Mb^c^	Gene panel (MSK-IMPACT)^e^	MuTect (10)	MSKCC, *JCO Precis Oncol* 2017 (*n* = 504)	(13)
	4.1 NS/Mb^b^	WGS	MuTect, Strelka (10, 22)	SU2C/PCF Dream Team, *Cell* 2018 (*n* = 101)	(9)
	44 NS/sample^c^^,^ ^e^	WES	Used Own Method	Fred Hutchinson CRC, *Nat Med* 2016 (*n* = 176)	(24)
	2.00 SNVs/Mb^c^	WES	Used Own Method	MCTP, *Nature* 2012 (*n* = 61)	(18)
	2.3 SNVs/Mb^b,d^	WGS	Freebayes, Pindel (25, 26)	UMichigan, *Cell* 2018 (*n* = 360)	(27)
	3.6 SNVs/Mb^c^	WGS	MuTect (10)	MSKCC/DFCI, SU2C/PCF Dream Team, *Cell* 2018 (*n* = 23)	(28)
	4.4 SNVs/Mb^c^	WES	MuTect (10)	SU2C/PCF Dream Team, *Cell* 2015 (*n* = 150)	(29)
	41 SNVs/sample^b^^,^ ^e^^,^ ^f^	WES	MuTect (10)	Multi-Institute, *Nat Med* 2016 (*n* = 114)	(30)
	98 SNVs/sample^c^^,^ ^e^	WGS	CaVEMan (31)	PELICAN Study, *Nature* 2015 (*n* = 10)	(31)

a *TCGA, The Cancer Genome Atlas; MSKCC, Memorial Sloan Kettering Cancer Center; DFCI, Dana-Farber Cancer Institute; PROGENY, PROstate cancer GENomic heterogeneitY; CPC-GENE, Canadian Prostate Cancer Genome Network; SAPCS, Southern African Prostate Cancer Study; MCTP, Medicaid Cancer Treatment Program; SU2C, Stand Up to Cancer; PCF, Prostate Cancer Foundation; CRC, Cancer Research Center; PELICAN, Project to ELIminate lethal CANcer*.

b *These are median values as reported*.

c *These are mean values as reported*.

d *Memorial Sloan Kettering-Integrated Mutation Profiling of Actionable Cancer Targets (MSK-IMPACT) is a targeted panel (32)*.

e *The human genome is ~3 Gb. The exome is about 1% of the genome, or ~30 Mb*.

f *Many samples in this cohort are neuroendocrine prostate cancer, rather than prostate adenocarcinoma*.

Prostate cancer that presents as *de novo* metastases, or reappears as macro-metastases following definitive prostatectomy/radiotherapy, is termed metastatic castration-sensitive prostate cancer (mCSPC) (34–36). Just as the pattern of individual mutations is similar between locoregional disease and mCSPC, so is the mean TMB (1.74 vs. 2.08 NS/Mb) (13). Likewise, a separate study showed that patients presenting with markedly elevated PSAs (≥15) and a biopsied MRI-positive primary lesion had no significant TMB difference compared to those found to have mCSPC disease (20). However, as the disease advances beyond mCSPC, so too does the TMB. Metastatic castration-resistant prostate cancer (mCRPC) can no longer be controlled with androgen ablation and is the most morbid and lethal clinical state. Several groups have noted that the TMB of mCRPC is accordingly increased (4.02 vs. 2.08 NS/Mb in mCSPC in one study) (Table 1) (13, 18, 27, 29, 31).

However, analyzing prostate tumor genomes solely via TMB misses many alterations, since the disease has a higher burden than many other cancers of chromosomal structural variants including insertions, deletions, inversions, translocations, gene-fusions, and tandem duplications (14, 37, 38). Locoregional prostate cancer cohorts have a highly variable structural variant burden, with a median of 19 structural variants per genome (range between 0 and 499, Table 2) (16). Like TMB, the structural variant burden correlates with Gleason score (17 in Gleason 6 disease compared to 22 in Gleason 7, *p* < 0.001) (Table 2) (16). The mCRPC cohorts have a much higher structural variant burden than in locoregional disease; median lies between 230 and 337 per study (Table 2), keeping in mind structural variant measurement is not standardized (9, 28, 29).

**Table 2:** Structural variant burden (SVB) in locoregional and mCRPC samples.

**Clinical state**	**Structural variant burden (SVs per sample)**	**Method of determining SVs**	**Cohort^a^**	**References**
Locoregional	19 SVs/sample^b^^,^ ^c^	Delly v0.5.5 (39)	CPC-GENE, *Nature* 2017 (*n* = 477)	(16)
mCRPC	230 SVs/sample^c^^,^ ^d^	SvABA (40); GROC-SVS (41); Long Ranger v2.1.2 (https://support.10xgenomics.com/genome-exome/software/pipelines/latest/using/wgs)	MSKCC/DFCI, SU2C/PCF Dream Team, *Cell* 2018 (*n* = 23)	(28)
	337 SVs/sample^b^^,^ ^c^	Manta v1.1.1 (42)	SU2C/PCF Dream Team, *Cell* 2018 (*n* = 101)	(9)

a *CPC-GENE, Canadian Prostate Cancer Genome Network; MSKCC, Memorial Sloan Kettering Cancer Center; DFCI, Dana-Farber Cancer Institute; SU2C, Stand Up to Cancer; PCF, Prostate Cancer Foundation*.

b *These are median values as reported*.

c *The human genome is ~3 Gb, of which the exome is about 1%, or ~30 Mb*.

d *These are mean values as reported*.

At the chromosomal level, mCRPC genomes frequently demonstrate polyploidy and/or aneuploidy. There are several NGS studies confirming that roughly ≥40% of mCRPC samples are triploid or more (9, 27, 43), a status itself associated with more translocations and SNVs (9). Regarding aneuploidy, about 75% of locoregional prostate cancer genomes have chromosomal arm-level alterations, and 23% possessed ≥5 arm-level alterations (44). As with TMB and structural variants, the degree of arm-level alterations correlates with Gleason score: only ~3% of Gleason 6 tumors have ≥5 arm-level alterations compared to ~40% of the very poorly differentiated Gleason 9–10 tumors. Even after adjusting for Gleason score, the degree of tumor aneuploidy predicted future lethal prostate cancer risk with a median follow-up of 15 years: patients with ≥5 arm-level alterations had a odds ratio for lethality of 5.34 (95% CI 2.18–13.1) compared with those with no aneuploidy (44).

The majority of NGS-based clinical testing involves targeted panels, rather than whole genome sequencing (WGS), making direct detection of some structural variants challenging. However, copy number alterations (CNA) of individual genes and genomic regions can be robustly detected, and they are an indirect measure of unbalanced structural variants and aneuploidy. The tumor CNA burden (TCB) is reported as the fraction of the measured genome with broad CNA. The median TCB of locoregional disease is ~7% of the genome altered (12, 13, 45). TCB differs statistically with age at diagnosis and Gleason score in a similar way to TMB: those diagnosed in their 40s have a median TCB of ~2% genome altered whereas those diagnosed in their 60s have ~9% altered (12). Gleason 6 tumors have median TCBs of ~1% genome altered, whereas Gleason ≥8 tumors have ~13% altered (12), consistent with other reports (46). The TCB of tumors confers considerable prognostic information (43, 45, 47, 48): it is significantly associated with biochemical recurrence (each 1% increase in TCB was associated with a 5–8% decrease in 5-years relapse-free survival) and future metastasis (45). This was independently verified (43), even after adjustment for Gleason score and TMB (47). TCB was also found to be associated with prostate cancer-specific death after adjustment for clinical parameters, such as CAPRA (CAncer of the Prostate Risk Assessment) score (49) or Gleason score (per 5% TCB, HR 1.49; 95% 1.30–1.70) (47). Unlike TMB, median TCB of mCSPC tumors is higher than locoregional disease (20–30% genome altered) and even higher in mCRPC tumors (cohort medians between 23 and ~38%) (9, 12, 13, 20, 24). TCB is negatively associated with overall survival in metastatic tumors in multivariate analysis even after adjustment for TMB (per 5% TCB, HR = 1.08; 95% CI 1.02–1.15) (47).

There is a subset of prostate cancer that emerges clinically in the treated mCRPC state, whereby the dominant metastatic histology is now either small-cell carcinoma or possesses neuroendocrine features. This treatment-emergent small-cell/neuroendocrine prostate cancer (t-SCNC) (30, 50) has both characteristic molecular and aggressive clinical features: there is an enrichment for RB1/TP53 genomic alterations and rapidly progressive visceral metastases. In one analysis, there were no statistically significant differences between TMB, TCB, or ploidy between t-SCNC vs. mCRPC adenocarcinoma (30). This is consistent with the postulation that neuroendocrine transdifferentiation may be driven substantially by epigenetic mechanisms (51).

Longitudinal analysis of prostate tumor genomes reveals further complexity in interpreting TMB and TCB, since mutational processes are dynamic, interrelated, can arise in a multi-focal setting, and evolve with different degrees of clonality (13, 31, 46, 52). One study reports that 40% of primary prostate tumors appear to be monoclonal i.e., one dominant clone is detected (46). The remaining 60% of primary tumors demonstrate subclonal populations (78% biclonal, 20% triclonal) originating from an ancestral clone. These polyclonal tumors have inferior clinical outcomes with respect to biochemical relapse following definitive prostatectomy/radiotherapy (HR 2.64; CI 1.36–5.15), and persisting after adjustment for standard clinical parameters and TCB (46). The polyclonality-related risk appears to be additive to those derived from the combination of TMB and TCB. Interestingly, triclonal tumors have a higher median PSA level at diagnosis (9.7 vs. ~7 for monoclonal/biclonal; *p* < 0.01), and polyclonal primary tumors are also more likely to develop metastases later on (OR = 4.01; *p* < 0.05). In polyclonal primary tumors, most of the TMB is truncal (median 87% of total SNVs) whereas the TCB is more evenly distributed between being truncal (55%) vs. branch-specific (45%). Moreover, the individual truncal CNAs are larger (median 11.5 vs. 6.5 Mb for branch-specific CNAs) and biased toward deletions (84% of all deletions are truncal). CNAs are also observed at chromosome ends, and the median telomere length of polyclonal tumors is 500 bp shorter than monoclonal tumors (46).

Altogether, the burden of genomic alterations correlates with key clinical information for prostate cancer patients. The TMB, structural variants, and TCB all tend to increase with advancing clinical state, Gleason score, and age. However, clonality analysis hints that how these mutational processes combine during tumor evolution is quite complex.

## Mutational Processes Underlying Prostate Cancer Genomic Alterations

Next, we examine the biologic processes that generate these genomic alterations, starting with SNVs. Comparison of tumor-derived patterns of SNVs within their trinucleotide context to pre-defined signatures (53) can suggest the underlying etiology; for example, cancers with known exogenous risk factors reveal robust signatures associated with tobacco or UV exposure. However, the majority of mCRPC tumors with intact DNA repair pathways reveal a robust signature that is endogenous, age-related and likely results from deamination of 5-methylcytosine to thymine at mCpG dinucleotides (COSMIC signature 1) (12, 54). If not repaired before DNA replication, this results in a permanent C > T transition. This signature is contributory in most cancer types and the frequency of associated SNVs correlates with the age at diagnosis in pan-cancer analysis, although not necessarily meeting statistical significance when analyzing each tissue individually (54). Nevertheless, the rate of prostate cancer SNVs attributed to this age-related signature loosely fits a slope of ~6 SNVs/Gb/year and may contribute to the increased TMB of patients diagnosed at older ages. Clonality analysis reveals that this age-related signature is most dominant early in prostate tumor evolution (46).

In prostate tumors possessing DNA repair defects, SNVs are associated with different dominant signatures. A recent NGS analysis of one cohort revealed 3% of genomes possess somatic DNA mismatch repair defects (MMRD) (55) caused by loss-of-function mutations in the canonical genes *MLH1, MSH2, MSH6*, or *PMS2*, and consistent with other cohorts (56–58). If one of the allelic mutations is germline, the patient has Lynch syndrome and possesses increased lifetime risk for several cancer types including prostate cancer (59, 60). These tumor genomes are 10- to 100-fold less likely to repair base pair substitutions prior to DNA replication, and their TMB is elevated (20–80 SNVs/Mb in mCRPC) although not necessarily as high as other MMRD cancer types (29). Analysis as above reveals dominant SNV signatures associated with MMRD, as expected (9, 27, 55, 61). MMRD tumors also possess high rates of indels (9), leading to higher instability of DNA microsatellite lengths, a way in which such tumors can be detected (55). MMRD tumors have distinct genomes from those that are MMR proficient: they are usually diploid, and have the lowest TCB (27). We further discuss MMRD tumors in the next section.

A third class of SNVs is observed in tumors with homologous recombination deficiency (HRD) from 6 to 20% of patients with either somatic or germline alterations of *BRCA1* or *BRCA2*, frequently biallelic (9, 13, 27, 29, 62). Since DNA homologous recombination coordinates the repair of double stranded DNA breaks, HRD not only results in a high TCB, but also a reliance on alternative error-prone DNA repair pathways (63) and a distinct dominant group of SNV signatures (9, 27). Accordingly, *BRCA*-mutant tumors in mCRPC possess the highest SNV rate among MMR proficient tumors (7.0 muts/Mb), in addition to higher TCB (9).

There are other SNV signatures observed to varying degrees in prostate tumors, some of which have not yet been associated with an etiology (27, 53). Moreover, SNVs are not evenly distributed throughout a given tumor's genome, but rather dependent on many interrelated factors, including the underlying mutational process, the timing of the locus within DNA replication, as well as whether the locus affects transcription and/or translation. The phenomenon of localized regions of SNV-based hypermutation is called kataegis, and is found in 23% of primary tumors (16); it is coincident with genomic instability, likely altered DNA repair (64), and enriched for deletions of the chromatin remodeler *CHD1* (33% of kataegis-positive tumors compared with only 11% of kataegis-null tumors) (16). Kataegis is associated with increasing Gleason score, and present in 40% of Gleason 4 + 3 tumors.

Just as specific processes lead to increased TMB, others lead to increased TCB. For example, *BRCA*-mutant tumors have markedly higher frequencies of copy number deletions as well as classic genomic “scars” due to their HRD (9). On the other hand, specifically in HR proficient tumors, chromothripsis can occur: evidence of “shattering” of regions in one or a few chromosomes followed by intrachromosomal reassembly in a stochastic manner, resulting in large numbers of both deletions and inversions (9). It is found in 20% of non-indolent primary prostate cancer samples (16) and 23% of mCRPC samples (9). Although the exact mechanism is unknown, there are some clear correlations: chromothripsis positive genomes are enriched for biallelic *TP53* loss (83% of chromothripsis positive tumors vs. 35% of chromothripsis null tumors), although this event is not likely sufficient to cause chromothripsis (9, 16). Others have noted a correlation between genomic loss of *CHD1* and chromothripsis (14). From a clinical standpoint, chromothripsis is associated with the primary tumor T-stage, but was not found to differ by age or Gleason grade (16).

About 5% of mCRPC cases have a significantly higher number of genomic tandem duplications, and 90% of these genomes have biallelic *CDK12* alterations (27, 28). In such cases there is a median of 150 tandem duplications per sample with a median duplicated region size of 1.3 Mb (28). Accordingly, such tumors possess large numbers of focal CNAs, and also have the highest gene-fusion burden (100 per tumor vs. 25 in other tumors), due directly to the genomic duplication phenomenon (27). *CDK12*-mutant tumors are usually diploid and trend toward mutual exclusivity from HRD biallelic *BRCA*-mutant tumors (9). Clonality analysis reveals that *CDK12* alterations are usually truncal; in these samples, the accompanying SNVs are more likely to occur after tandem duplication than before, and in many cases in branch-specific subclones (28). It is unknown whether *CDK12* alterations directly cause the tandem duplications, or are merely associated with it, but there is evidence to support the former (28).

Finally, some mutational processes occur without directly affecting TMB or TCB. The most common gene fusions in prostate cancer occur between androgen-driven upstream elements of genes like *TMPRSS2*, and oncogenic ETS transcription factors like *ERG*, and are present in up to 50–60% of men of European descent (65). The underlying chromosomal rearrangements that cause such gene fusions are initially balanced, frequently complex and involve multiple chromosomes in a phenomenon termed chromoplexy (14). Some degree of chromoplexy is present in 50–90% prostate tumors (9, 14). Moreover, in tumors possessing ETS gene fusions, the chromoplexy has more than double the number of interchromosomal rearrangements compared to ETS fusion-null tumors (14). There is evidence of successive rounds of chromoplexy occurring, for example initially leading to ETS fusion formation, and then subsequently to inactivation of tumor suppressor genes. It is not known exactly how chromoplexy occurs; there is no enrichment for *TP53* mutations in such tumors, but the process may be related to androgen-related chromatin configuration (9, 14). Notably, the small cohort of South African men possessed lower frequencies of larger genomic rearrangements, such as chromothripsis and chromoplexy, and lower frequencies of ETS gene fusions, than the comparable cohort with European ancestry (23).

In summary, we have just begun to understand the processes that contribute to the burden of prostate cancer genomic alterations. SNVs possess distinct mutational signatures including those associated with aging and DNA repair defects; moreover, many tumor genomes have localized hypermutated regions. Complex chromosomal alterations, such as chromothripsis, tandem duplication, and chromoplexy tend to stratify by specific alterations in *TP53, CHD1, CDK12*, and *BRCA1/2* and underlie many CNAs and fusion events, such as the canonical *TMPRSS2*-*ERG* fusion.

## Prostate Tumor Mutation Burden, DNA Repair Defects, and Therapeutic Response

The initial trials of immune checkpoint inhibitors in unselected prostate cancer patients (66–68) demonstrated no global clinical benefit in prostate cancer. Nevertheless, interest in such therapies remained strong, given case reports of impressive and durable responses among individual prostate cancer patients (69, 70). Experiences from other tumor types illuminated patient subtypes that may derive clinical benefit from existing therapies. Efforts to identify a specific predictive biomarker, such as PD-L1 expression have been challenging (71, 72); however, the association with global genomic processes has been clear. Patients with non-small cell lung cancers (73), bladder cancers (74), and melanoma (75–77) that have a high TMB derive increased clinical benefit to immune checkpoint inhibitors compared to those with low TMB. There are markedly different numerical thresholds of what constitutes a high TMB, with the highest quintile within a given histology usually being associated with longer overall survival when treated with immune checkpoint inhibitors (78). Increased non-synonymous mutations and indel frameshifts lead to increased neoepitopes within MHC Class I-loaded peptides and it is hypothesized these serve as neoantigens in the context of immunotherapy (79–84). Ongoing prostate cancer immunotherapy trials are now beginning to incorporate TMB analyses (85).

As described above, the prostate tumors with the highest TMB are those with MMRD. A series of trials treating patients with MMRD tumors with pembrolizumab, regardless of histology, reported a 53% objective radiographic response rate, and a 21% complete response rate (86). This led to the FDA approval of pembrolizumab for any MMRD metastatic/unresectable solid tumors and reinforces the importance of testing such prostate cancer patients for MMRD. In a recent study of mCRPC patients with MMRD tumors and treated with immune checkpoint inhibitors, 55% achieved a PSA response >50%, and 45% of patients had durable clinical benefit (55). A smaller study revealed that three out of four patients with MMRD tumors achieved soft tissue tumor responses upon treatment with immune checkpoint inhibitors (56). In a separate large analysis of mCRPC samples, MMRD tumors were predicted to have median neoantigen burdens of ~10,000 vs. 1,000 in MMR proficient tumors (27). Approximately 10% of these are further predicted to be “strong binders” of MHC Class I. MMRD prostate tumors were also found to have high degrees of immune infiltration (20, 27), the highest number of T-cell clonotypes, and the highest percent of expanded T-cell clones (27). Other studies have showed a complex relationship between predicted neoantigen load with immune infiltration (20), as well as considerable heterogeneity of tumor T-cell infiltration in MMRD cases (87). The exact mechanism of how predicted neoantigens stimulate a clinically-relevant immunologic response, and how this might inform the next generation of immunotherapies remains an active area of study (77).

Prostate tumors with MMRD may have other unique molecular and biological features, compared to MMR proficient disease. One case series reported an enrichment of MMRD among ductal adenocarcinoma of the prostate, a rare aggressive subtype of prostate adenocarcinoma (about 3% incidence, compared to the common acinar adenocarcinoma) with poor prognosis (56, 57, 88). Ultimately, it is important to understand the natural history of prostate cancer patients with MMRD tumors, particularly prior to any potential treatment with immune checkpoint inhibitors. One study of patients with recurrent disease reports a longer progression free survival following androgen-deprivation therapy when MMRD is detected (median 66 months compared to only 27 months in MMR proficient cases), as well as longer responses to first-line ARSI agents when used (56). On the other hand, among patients with clinically aggressive tumors (56% having metastatic disease at diagnosis), a different retrospective study of clinically aggressive CRPC noted the median overall survival for the MMRD cases was significantly shorter (3.8 years from androgen ablation) than MMR proficient groups (7.0 years), in both univariate and multivariate analysis (87). The studies above cannot be directly compared, but perhaps the biologic context is key to interpreting the clinical relevance of MMRD.

Beyond MMRD, prostate cancers with other DNA damage repair defects are being explored for their responses to immunotherapies. Because *CDK12*-mutant tumors have increased rates of gene fusions, they possess higher predicted neoantigen burdens (median ~2,000) than other MMR proficient tumors (27). They may also possess high degrees of T cell infiltration and expanded T cell clones. These findings have led to a Phase II trial evaluating the efficiency of combination nivolumab plus ipilimumab in mCRPC patients with *CDK12*-mutant tumors (89). There are also several immune checkpoint inhibitor therapy combinations being explored, such as one in which the second agent, a PARP inhibitor, alters how the genome repairs itself (90). Interestingly, a recent phase Ib/2 study showed some interesting clinical responses to the combination of pembrolizumab and olaparib, despite no BRCA mutations being detected in the biopsies tested (91). Whether this clinical response is due undetected HRD, or whether the PARP inhibitor synergizes with the immune checkpoint inhibitor by altering the presented neoepitopes or an unknown mechanism remains to be determined.

While high TMB, particularly in the context of altered DNA repair, is important regarding successful immune checkpoint therapy in prostate cancer, it is certainly not the whole story. Roughly half of MMRD prostate tumors do not exhibit substantial clinical responses to such therapy despite relatively high TMB (55). Moreover, when clinical responses are observed, the TMB is often lower compared to that observed in other MMRD cancer types e.g., in a preplanned interim analysis of a small phase II mCRPC study of combination nivolumab plus ipilimumab, responses were observed in tumors above a modest TMB threshold (85). Identifying other genomic factors that modify response to immune checkpoint inhibitors and determining whether they map to specific genes and/or global processes, remains an active area of investigation. Due to NGS-based analysis of patients' tumors, we are just beginning to obtain a comprehensive snapshot of the prostate tumor genome in differing clinical states. A deeper understanding whether and how global genomic measures, such as TMB, TCB, gene-fusion burden and clonality affect responses to targeted and immuno-therapies will help us shape future prostate cancer investigations.

## Author Contributions

MR and RB drafted, revised, and approved the submitted version.

### Conflict of Interest

The authors declare that the research was conducted in the absence of any commercial or financial relationships that could be construed as a potential conflict of interest.
